# Cardiovascular Disease and Hip Fracture among Older Inpatients in Beijing, China

**DOI:** 10.1155/2013/493696

**Published:** 2013-07-01

**Authors:** Beibei Xu, Ling Han, Hui Liu, Jing Wang, Xiao-Yuan Bao, Han-Xu Xi, Leping Zhao, Guo-Pei Yu

**Affiliations:** ^1^Medical Informatics Center, Peking University, 38 Xueyuan Rd, Haidian District, Beijing 100191, China; ^2^Yale University School of Medicine Program on Aging & Internal Medicine, New Haven, CT 06511, USA; ^3^New York College of Medicine, Valhalla, NY 10595, USA

## Abstract

*Objectives*. To examine the associations between cardiovascular disease (CVD) and hip fracture and to determine if these associations are attributable to hypertensive disease. *Methods*. Data were obtained from 2006–2010 hospitalization summary reports of 31 tertiary hospitals in Beijing, China. This study included 864,408 inpatients aged ≥55 years. Occurrence rate of hip fracture was based on the first-listed ICD-10 codes (S72.0, S72.1, and S72.2) and of CVD as comorbidities were based on the second- to the eighth-listed ICD-10 codes (I00–I99). *Results*. The occurrence rate of hip fracture is 53% higher among older inpatients with a diagnosis of CVD than those without (RR = 1.53, 95% CI 1.47–1.60). Those with hip fracture were more likely to have hypertensive or cerebrovascular disease, with the risk ranging from 1.34 to 1.70. Compared with those without hip fracture, the occurrence rate of overall CVDs increased by 80%, 83%, and 16% among hip fracture patients aged 55–64, 65–79, and ≥80 years. In addition, hypertensive disease did not modify the association between cerebrovascular disease and hip fracture. *Conclusion*. CVD was positively associated with hip fracture, and the associations observed in this sample of Chinese inpatients were similar to those reported from cohort studies conducted in the European populations.

## 1. Introduction

Hip fracture, as a serious consequence of osteoporosis, has been associated with multiple comorbidities including cardiovascular disease (CVD) among older adults [1–4]. The incidence rate of hip fracture was about 13 per 1000 person-year after a diagnosis of CVD during the 20 years of followup from the age of 50 years reported by a European study [1]. Researchers have suggested common etiologic mechanisms for CVD and hip fracture, two independent age-related disorders previously considered [1, 5–8]. Bone mineralization and vascular calcification shared common pathologic pathways and medications, including cholesterol-lowering stains and bisphosphonates which have been shown to affect both atherosclerosis and osteoporotic fractures [1, 5, 6]. A large Swedish twin study suggested a genetic predisposition for both vascular and bone health [1]. Although this genetic explanation remains at large, specific attention has been devoted to hypertension, which is believed to predispose individuals not only to heart disease and stroke but also to bone mineral loss [7, 8]. 

The associations of hip fracture with CVD were essentially derived from studies conducted in European populations [1]. There has been a lack of comparable data in Asian populations, particularly among Chinese. Over the past 10 to 20 years, the mainland China has seen a dramatic increase in the incidence of hip fracture [9]. For instance, the age-specific rates of hip fracture has increased by 2.76-fold and 1.61-fold among women and men aged ≥50 years from the years 1990–1992 to 2002–2006 in Beijing, China [9]. The incidence of hip fractures is predicted to continue rising as a result of a fast-aging population [10]. Meanwhile, CVD has become the number one cause of hospitalization and mortality in China since the 1990s and is anticipated to continue to dominate the all-cause mortality [11]. 

In this study, using data from 864,408 hospitalized patients from 31 tertiary hospitals in Beijing, the capital city of China, we sought to investigate whether there is an independent association between subtypes of CVD and hip fracture and to examine whether the association is attributable to hypertensive disease. We hypothesized that patients with hip fracture had an increased risk of CVD in comparison with those without hip fracture, and such an association could be reliably estimated by a large hospital-based study.

## 2. Methods

### 2.1. Data Source

Data were obtained from 2006–2010 hospitalization summary reports (HSR) in 31 tertiary hospitals in Beijing, China. The HSRs were electronically submitted to the Beijing Municipal Health Bureau, through a centralized health information system, in accordance with the administrative requirements of the Ministry of Health. The medical information recorded on the HSR includes basic demographics, date of admission and discharge, eight discharge diagnoses in Chinese and corresponding ICD-10-CM codes (1 principal and 7 supplementary diagnoses), treatments (mainly surgical information including date, coding, anesthesia, and surgeon), outcome of hospitalizations (survival status, drug allergy, and hospitalization infection), and financial costs. Our study included hospitals with 500 or more beds in Beijing, China. Data from military hospitals were not available and special hospitals (cancer, infectious disease, gynecology, and chest hospitals) were excluded for this study because too few patients were diagnosed as having CVDs or hip fracture. This study is considered exempt since it used data collected for an administrative purpose without any personal identifiers.

### 2.2. Study Sample and Participants

 We first identified patients with hip fracture (ICD-10-CM codes: S72.0, S72.1 and S72.2) by the first-listed diagnosis and then captured and defined patients with CVD as comorbidities by the second- to the eighth-listed diagnoses. The subtypes of CVDs including hypertensive diseases (I10–I15), ischemic heart disease (I20–I25), other heart diseases (I00–I09, I26–I51), cerebrovascular disease (I60–I69), and other circulatory diseases (I70–I89, I95–I99) were analyzed. 

For comparison purposes, we also identified those who did not have diagnoses on hip fracture in the first-listed code. To remove the influence of other diseases on the associations, we excluded patients with a diagnosis of CVD or influenza and pneumonia (J09–J18) in the first-listed code from the comparison group. Cardiovascular events among the comparison group were captured by the same method of the second- to the eighth-listed diagnostic codes. 

For this study, cardiovascular events were defined as accompanied diseases and therefore risk factors of hip fracture. The unit of analysis was an individual patient, not a hospitalization, and the first hospitalization of patient, if there were a few hospitalizations, was served as the index patient for analysis. All hospitalized patients aged ≥55 years were included in this study, and the final sample size is 864,408. 

### 2.3. Statistical Analysis

To compare the difference in the proportion of diagnosis of CVD between patients with and without hip fracture, SAS PROC GENMOD's log-binomial regression capability was used to estimate the age-, sex-, and comorbidity-adjusted rate ratios (RRs) and 95% confidence intervals (CIs) [12]. 

In the models, the effect of CVD on the risk of hip fracture was assumed to be independent. For each CVD subtype, covariates containing a dependent variable (the probability of having a hip fracture) and independent variables such as age (55–64, 65–79, and ≥80 years), sex (male and female), and numbers of comorbidities (1-2, 3-4, ≥5) was established. A rate ratio > 1 with *P* value <0.05 indicated a statistically significant association of a CVD diagnosis with hip fracture. The greater the amount of RRs, the stronger the association of CVD with hip fracture. Instead of using matching procedure originally designed to raise the study validity while diminishing the statistical power of results, the procedure of multivariate analysis with larger sample size in comparison group has been suggested to handle confounding situations [13]. Statistical analyses were performed using STATA 12.0 (StataCorp LP, College Station, TX, USA). 

## 3. Results

This analysis included 13,071 hospitalized patients with hip fracture and 851,337 without hip fracture as controls (Table 1). Patients with hip fracture differed from those without hip fracture in the distributions of gender, age, and number of diagnosis, with higher proportion of female (63.9% versus 49.1%) and patients aged ≥80 years (35.8% versus 12.3%). In addition, patients with hip fracture had a lower mortality rate (1.0% versus 3.3%), but a longer length of hospital stay (19 days versus 12 days), higher surgical rate (89.6% versus 60.2%), and higher cost per hospital stay (37,757.6 CNY versus 10,810.3 CNY). 

Table 2 shows the associations between CVD and hip fracture. Approximately 54.3% of 13,071 older patients with hip fracture had a diagnosis of CVD during hospitalization, while the proportion of diagnosis of CVD was 49.0% among 851,337 patients without hip fracture. Overall, if older patients had a supplementary diagnosis of CVD, the risk of hip fracture increased by 53% (RR = 1.53, 95% CI 1.47–1.60, *P* < 0.001), after adjusting for age, gender, and number of comorbidities. In the cardiovascular subtype analysis, the rate of hypertensive disease was found to be high, 40% and 35.7%, respectively, regardless of patients with or without hip fracture. In contrast, the rates of ischemic heart disease and cerebrovascular disease were 14.9% and 16.6% among patients with hip fracture, while the rates of these diseases were 14.5% and 12.7% among patients without hip fracture. The adjusted rate ratios for hypertensive diseases, ischemic heart disease, and cerebrovascular disease were 1.34 (95% CI 1.29–1.40, *P* < 0.001), 1.05 (95% CI 1.00–1.11, *P* = 0.062), and 1.70 (95% CI 1.62–1.79, *P* < 0.001) comparing patients with hip fracture to those without hip fracture. 

Gender-specific associations between CVD and hip fracture are listed in Table 3. The risk of hip fracture increased by 48% (RR = 1.48, 1.40–1.56, *P* < 0.001) among male patients and by 63% (RR = 1.63, 1.52–1.76, *P* < 0.001) among female patients if hip fracture patients had a supplementary diagnosis of CVDs. The adjusted rate ratios of hypertensive disease and cerebrovascular disease were significantly high, ranging from 1.33 to 1.92, among two gender groups, while that of ischemic heart disease was relatively low, about 1.13 (95% CI 1.06–1.21, *P* < 0.001) among male patients, and not significant among female patients. 

Age stratified associations of CVD with hip fracture are shown in Table 4. Compared to those without hip fracture, the proportion of CVD increased by 80% (RR = 1.80, 95% CI 1.60–2.04, *P* < 0.001), 83% (RR = 1.83, 1.71–1.95, *P* < 0.001), and 16% (RR = 1.16, 1.08–1.24, *P* < 0.001) among patients with hip fracture aged 55–64 years, 65–79 years, and ≥80 years. Overall, the occurrence rate of hypertensive disease was almost two-fold or higher than that of ischemic heart disease and cerebrovascular disease among all three age groups. Hip fracture was significantly associated with hypertensive diseases, ischemic heart disease, and cerebrovascular disease among patients aged between 55 and 79 years, but such association attenuates among those aged ≥80 years. 

To examine if hypertension affects the associations between other CVD subtypes and hip fracture, we further analyzed data by stratifying two groups of patients with and without hypertension. As shown in Table 5, the prevalent rates of all cardiovascular subtypes were higher among hypertensive patients than among nonhypertensive patients, regardless of hip fracture or non-hip fracture patients. Hip fracture patients were more likely to have a diagnosis of ischemic heart disease (RR = 1.16, 95% CI 1.06–1.26, *P* < 0.001) if they had no diagnosis of hypertensive diseases, while such an association was not seen among hypertensive patients. Nevertheless, hypertensive diseases did not seem to modify the association between cerebrovascular disease and hip fracture, in which the diagnosed proportion of cerebrovascular disease increased by 71% (RR = 1.71, 1.58–1.86, *P* < 0.001) among hip fracture patients without hypertension and by 66% (RR = 1.66, 1.55–1.77, *P* < 0.001) among those with hypertension.

## 4. Discussion

Our study observed a significant association between CVD and hip fracture among older hospitalized patients in Beijing, China, with a 53% increase in the risk of hip fracture. This association seems to be accounted for mainly by patients of 55 to 79 years old, and by specific subtype of CVD, the cerebrovascular disease, as evidenced by a more than 2-fold increase in the estimated hip fracture risk. In addition, hypertensive disease did not modify the association between cerebrovascular disease and hip fracture. 

Our findings were similar to several previous reports from Sweden and the US populations, which reported a significant association between CVD and risk of hip fracture [1, 14]. However, the strength of association observed in our study was somewhat lower than that of European studies. Although it is possible that such discrepancy may result from the methodological difference. More likely the other fundamental factors such as the different rate of occurrence of osteoporosis may also play a role. It has been suggested that osteoporotic fracture occurs approximately two- to three-fold lower in Chinese than in many European countries, and one-fold lower in US Asians than in US Caucasians [15]. 

The similar findings between our study and previous studies included a weak association with ischemic heart disease and a strong association with stroke. In a prospective study in Sweden, the risk of hip fracture was found to increase by one-fold after a diagnosis of an ischemic heart disease and by four-fold after a stroke [1]. In our study, the presence of cerebrovascular disease increased the risk of hip fracture by 70%, while no statistically significant association was found between hip fracture and ischemic heart disease among older inpatients aged ≥55 years. 

Researchers suggested that the different strengths of association observed in ischemic heart disease and stroke may be explained by the role of hypertensive disease [7]. High blood pressure may insidiously damage brain structures related to gait control and balance and thus could predispose to falls and subsequent fractures. This is supported by the findings that the white matter lesions of the brain were independently associated with an almost three-fold increase in the risk of hip fractures in an Italian prospective study [16]. However, we noted that the association between cerebrovascular disease and hip fracture always existed regardless of whether the impact of hypertension is involved. Our results suggest that the stroke may be the primary driving force of the observed association between CVD and hip fracture and, hence, may itself serve as a stand-alone risk factor for hip fracture.

We noted the sex and age differences in the association between CVDs and hip fracture risk. The presence of cerebrovascular disease was associated with a 30% higher risk of hip fracture for male patients than for female patients. Although the reason for the gender effect is unclear, our result is consistent to previous reports that men had a higher risk to fall after a stroke than women [17]. With regard to the age difference, the associations between CVDs and hip fracture were seen among patients aged 55–79 years but diminished among those aged ≥80 years. The significantly strong associations of CVDs among patients aged 55–79 years may reflect more physical activities among this population. In other words, people with CVDs who survived until 80 years or older tend to be physically inactive and thus have less chance to fall. Certainly, it is also likely that when CVDs and hip fracture both occur, patients aged at 80 years or older may have already died before they are admitted to the hospital. 

Our data from the 31 hospitals are unique because of the large sample size and comprehensive representation of the entire hip fracture patients in the top-ranked hospitals in Beijing. Our study design is quite distinctive, not only including large numbers of hip fracture patients and comparisons but also using all hospitalized patients without non-hip fracture as comparisons. In this study, we assumed that the presence of CVD was a random phenomenon in non-hip fracture hospitalized patients, and it was measured reliably in a very large population including approximately one million patients, just as in the general population. Since our findings of the association between CVD and hip fracture are significantly positive, such assumption on non-hip fracture hospitalized patients should have been valid. To our knowledge, this study is the first to carry on a hospital-based two group comparison study with nearly one million patients for comparisons to investigate the associations between CVDs and hip fracture. 

However, our results depend on the quality of the HSR data. We consider that the overall quality of the HSR data is quite high. This is because all hospitals we analyzed are top-ranked hospitals in the capital city of China and have a high reputation for their quality in all aspects of healthcare, including diagnosis, treatment, hospital management, coding, and electronic medical record systems. Secondly, the HSR data were required by the Beijing Municipal Health Bureau for computing allocations of financial resources and evaluating hospital performance. As a result, each hospital had been committed to deliver high quality data. 

Our study has some limitations. Firstly, despite quality controls, the HSR data are still subject to measurement errors, which include errors due to incomplete or inaccurately recorded information on summary reports and processing errors. There is also a possibility of misclassification of disease in coding, especially for some subtypes of cardiovascular disease. However, the misclassification of coding, if exists, should occur randomly, unlikely depending on the diagnosis of hip fracture; thus it may not attenuate our estimates on hip fracture analysis. Secondly, the HSR data are restricted to hospitalized patients; we therefore could not analyze patients with hip fracture outside hospitals, including those not admitted or being discharged. But we think that the etiology should be the same for all patients with hip fracture, regardless of being hospitalized or not hospitalized. Thirdly, the associations of CVD and hip fracture reported by our study may be underestimated because the health status of hospitalized patients is generally worse than that of the general population. As a result, the comparison group may have a higher occurence rate of CVDs and reduce the strength of association. Fourthly, although we have adjusted to several factors, it is possible that residual confounding effects of other unknown factors remain which could attenuate the positive association between CVDs and hip fracture. 

We suggest effective preventions for falling for older patients with CVD, particularly for those who were ever diagnosed by cerebrovascular diseases. To reduce the occurrence of hip fracture from falling, older cardiovascular patients should be screened for osteoporosis and treated if necessary. Medication should be prescribed for those older patients at long-term bed rest or those active patients with substantial bone loss shown in medical examinations. On the other hand, control of hypertension, proper physical activity, and improved nutrition are also likely to be important strategies for reducing the increased occurrence of hip fracture in China. 

## Figures and Tables

**Table 1:** Characteristics of inpatients with and without hip fracture.

	Hip fracture (*n* = 13,071)	Non-hip fracture (*n* = 851,337)
	No (%)	No (%)
Gender		
Male	4,720 (36.1)	476,719 (52.1)
Age (y)		
55–64	1,616 (12.4)	334,067 (36.5)
65–79	6,770 (51.8)	468,597 (51.2)
≥80	4,685 (35.8)	112,285 (12.3)
Number of diagnosis		
1-2	5,565 (42.6)	346,240 (37.8)
3-4	4,232 (32.4)	229,510 (25.1)
≥5	3,274 (25.1)	339,199 (37.1)
No. of surgery (%)	11,710 (89.6)	551,197 (60.2)
No. of death (%)	130 (1.0)	30,356 (3.3)
Median length of stay (days)*	19 (13, 25)	12 (7, 20)
Median cost per stay (CNY)*	37,757.6 (23,801.5, 48,284.7)	10,810.3 (6,040.7, 20,604.2)

*Numbers in brackets are the 25th and 75th percentile.

**Table 2:** Rate ratios of cardiovascular diseases associated with hip fracture among patients aged ≥55 years.

	Hip fracture (*n* = 13,071)		Non-hip fracture (*n* = 851,337)		Adjusted rate ratio (95% CI)*	*P* value
	No.	%	No.	%		
Total	7,101	54.3	448,397	49.0	1.53 (1.47, 1.60)	<0.001
Hypertensive diseases (I10–I15)	5,233	40.0	326,521	35.7	1.34 (1.29, 1.40)	<0.001
Ischemic heart disease (I20–I25)	1,948	14.9	132,237	14.5	1.05 (1.00, 1.11)	0.062
Other heart diseases (I00–I09, I26–I51)	1,070	8.2	100,515	11.0	0.73 (0.68, 0.78)	<0.001
Cerebrovascular disease (I60–I69)	2,175	16.6	115,896	12.7	1.70 (1.62, 1.79)	<0.001
Other circulatory diseases (I70–I89, I95–I99)	650	5.0	55,624	6.1	1.09 (1.01, 1.18)	0.034

*Rate ratios and 95% confidence intervals were adjusted for sex, age, and number of diagnosis.

**Table 3:** Rate ratios of cardiovascular diseases associated with hip fracture by gender.

	Hip fracture		Non-hip fracture		Adjusted rate ratio (95% CI)*	*P* value
	No.	%	No.	%		
Female	2,516	53.3	236,030	49.5	1.48 (1.40, 1.56)	<0.001
Hypertensive diseases	1,724	36.5	163,508	34.3	1.33 (1.27, 1.40)	<0.001
Ischemic heart disease	590	12.5	71,443	15.0	1.13 (1.06, 1.21)	<0.001
Other heart diseases	375	7.9	57,498	12.1	0.76 (0.70, 0.83)	<0.001
Cerebrovascular disease	945	20.0	69,102	14.5	1.58 (1.49, 1.69)	<0.001
Other circulatory diseases	254	5.4	30,842	6.5	1.06 (0.96, 1.18)	0.237
Male	4,585	54.9	212,367	48.5	1.63 (1.52, 1.76)	<0.001
Hypertensive diseases	3,509	42.0	163,013	37.2	1.35 (1.26, 1.44)	<0.001
Ischemic heart disease	1,358	16.3	60,794	13.9	0.93 (0.84, 1.02)	0.126
Other heart diseases	695	8.3	43,017	9.8	0.68 (0.61, 0.76)	<0.001
Cerebrovascular disease	1,230	14.7	46,794	10.7	1.92 (1.77, 2.08)	<0.001
Other circulatory diseases	396	4.7	24,782	5.7	1.13 (0.99, 1.28)	0.067

*Rate ratios and 95% confidence intervals were adjusted for age and number of diagnosis.

**Table 4:** Rate ratios of cardiovascular diseases associated with hip fracture by age groups.

	Hip fracture		Non-hip fracture		Adjusted rate ratio (95% CI)*	*P* value
	No.	%	No.	%		
55–64 years	594	36.8	116,406	34.9	1.80 (1.60, 2.04)	<0.001
Hypertensive diseases	456	28.2	87,432	26.2	1.61 (1.43, 1.82)	<0.001
Ischemic heart disease	90	5.6	20,231	6.1	1.43 (1.14, 1.79)	0.002
Other heart diseases	50	3.1	15,592	4.7	0.98 (0.73, 1.31)	0.881
Cerebrovascular diseases	172	10.6	22,706	6.8	2.94 (2.47, 3.52)	<0.001
Other circulatory diseases	70	4.3	16,743	5.0	1.31 (1.02, 1.68)	0.033
65–79 years	3,819	56.4	253,466	54.1	1.83 (1.71, 1.95)	<0.001
Hypertensive diseases	2,897	42.8	186,507	39.8	1.47 (1.39, 1.55)	<0.001
Ischemic heart disease	975	14.4	78,110	16.7	1.18 (1.10, 1.27)	<0.001
Other heart diseases	506	7.5	56,971	12.2	0.85 (0.77, 0.93)	0.001
Cerebrovascular disease	1,261	18.6	65,809	14.0	2.34 (2.18, 2.50)	<0.001
Other circulatory diseases	308	4.6	30,403	6.5	0.94 (0.83, 1.05)	0.281
≥80 years	2688	57.4	78,525	69.9	1.16 (1.08, 1.24)	<0.001
Hypertensive diseases	1,880	40.1	52,582	46.8	1.12 (1.05, 1.19)	0.001
Ischemic heart disease	883	18.9	33,896	30.2	0.93 (0.86, 1.01)	0.072
Other heart diseases	514	11.0	27,952	24.9	0.66 (0.60, 0.73)	<0.001
Cerebrovascular disease	742	15.8	27,381	24.4	1.09 (1.00, 1.18)	0.042
Other circulatory diseases	272	5.8	8,478	7.6	1.20 (1.07, 1.36)	0.003

*Rate ratios and 95% confidence intervals were adjusted for sex and number of diagnosis.

**Table 5:** Rate ratios of cardiovascular diseases associated with hip fracture by hypertensive disease.

	Hip fracture		Non-hip fracture		Adjusted rate ratio (95% CI)*	*P* value
	No.	%	No.	%		
With hypertensive diseases	5233	100	326521	100	N/A	N/A
Ischemic heart disease	1,264	24.2	85,416	26.2	0.96 (0.90, 1.03)	0.279
Other heart diseases	548	10.5	52,720	16.2	0.66 (0.60, 0.72)	<0.001
Cerebrovascular disease	1,399	26.7	74,062	22.7	1.66 (1.55, 1.77)	<0.001
Other circulatory diseases	286	5.5	28,278	8.7	0.84 (0.74, 0.95)	0.004
Without hypertensive diseases	1868	23.8	121,876	20.7	1.53 (1.44, 1.63)	<0.001
Ischemic heart disease	684	8.7	46,821	8.0	1.16 (1.06, 1.26)	0.001
Other heart diseases	522	6.7	47,795	8.1	0.88 (0.80, 0.97)	0.010
Cerebrovascular disease	776	9.9	41,834	7.1	1.71 (1.58, 1.86)	<0.001
Other circulatory diseases	364	4.6	27,346	4.7	1.45 (1.30, 1.61)	<0.001

*Rate ratios and 95% confidence intervals were adjusted for sex, age, and number of diagnosis.
